# Dr. Charles Smith

**Published:** 1912-05

**Authors:** 


### OBITUARY

---

Dr. Charles Smith of Campbell, Steuben Co., died suddenly March 29, 1912 of what was termed acute indigestion. He was born in Cameron, a neighboring town, 47 years ago. He was a graduate of the University of Buffalo, 1890.

